# A qualitative study assessing allied health provider perceptions of telepractice functionality in therapy delivery for people with disability

**DOI:** 10.1111/hex.13988

**Published:** 2024-02-13

**Authors:** Cloe Benz, Jaya Dantas, Mai Welsh, Richard Norman, Suzanne Robinson, Delia Hendrie

**Affiliations:** ^1^ School of Population Health Curtin University Bentley Western Australia Australia; ^2^ Rocky Bay Mosman Park Western Australia Australia; ^3^ Deakin Health Economics, Institute for Health Transformation Deakin University Melbourne Victoria Australia

**Keywords:** accessibility, allied health, disability, PPI, qualitative, telepractice, teletherapy

## Abstract

**Introduction:**

Telepractice service delivery of allied health interventions to people with disability can potentially reduce access barriers and improve service equity. However, questions remain regarding telepractice functionality for people with disability. This study addressed questions related to how allied health clinicians and managers perceive telepractice as functioning in the provision of therapy services to people with disability.

**Methods:**

Thirteen interviews of allied health clinicians and managers from across Australia were conducted between 21 November and 22 February via MS teams. Qualitative methodology and critical realist theoretical paradigm underpin the study. Data analysis was completed using a reflective thematic analysis method and five themes were generated and described utilising an analytic metaphor.

**Results:**

The study themes were described in relation to a shopping for shoes analytic metaphor and the five themes included (1) a shoe for every foot, (2) planned purchases, (3) shoe on the other foot, (4) you need both shoes and (5) help choosing their shoes. In summary, the function of telepractice fits differently for each individual, similar to pairs of shoes.

**Conclusions:**

Telepractice has its own strengths and weaknesses and isn't a direct substitute for in‐person sessions, much like left and right shoes are similar but not the same. The results support participant perceptions that telepractice functions best as an adjunct to in‐person sessions through a flexible hybrid delivery model in the provision of therapy services to people with a disability. A strategy for improving perceived usefulness may involve positioning telepractice as unique with strengths and weaknesses, not replacing in‐person care.

**Patient or Public Contribution:**

The paper forms part of a larger codesign process which included customer and carer participants throughout the design and planning of the project, inclusion of a peer researcher, and the selection of the analytic metaphor including in the findings of this article production.

## 1 INTRODUCTION

The National Disability Insurance Scheme (NDIS) is an Australian federal government‐funded programme (roll out completed July 2020) which provides personalised funding plans to people with significant or permanent disability to access supports via a goal‐based model.^1^ Services available to access under the NDIS include allied health (e.g., physiotherapy, occupational therapy, speech pathology, dietetics), behaviour support and nursing care.^2^ NDIS participants are able to utilise their funding plan within a fixed term period (1–3 years) to receive services within categories; with each service (e.g., occupational therapy) allocated a maximum charging rate.^3^

A cornerstone policy of NDIS implementation in Australia is choice and control for participants,^4^ which supports the United Nations Convention on the Rights of Persons with Disabilities and challenged disability service providers to include clients in all decisions.^5^ Offering genuine choice and control requires a concerted effort to provide appropriate information, support in decision making, and trials of available options to understand how each choice may impact them.^6^ Additionally, providing a service which is designed to be universally accessible is necessary to facilitate genuine choice.

Universal design is a principle which promotes equitable access, which is often referred to in the context of the built environment (access to places) but can be applied to services, it considers how barriers may be created which are disabling for specific people.^7^ Distribution of therapy services to the disability community both within and across countries can be extremely variable and are significantly impacted by location thus causing inequitable access. In Australia, there is an average population of 3.33 people/km^2^ (world average of 62 people/km^2^)^8^ ranking it the fourth least densely populated country^9^ in the world. Staff recruitment and retention issues, waiting lists, travel time and lack of choice in service providers are a cause of disparity and remain ongoing challenges across different areas of Australia.^10^

Telepractice service delivery of allied health interventions to people with disability potentially provides an excellent opportunity to reduce access barriers and improve service equity and access.^11^ Telepractice is defined as the use of telecommunications technology to deliver clinical services remotely to a client or carer for the purpose of assessment, intervention, consultation and supervision.^12^

Whether telepractice service delivery is functional for recipients and clinicians may depend on what alternatives are available or if local infrastructure (e.g., internet connection quality) can support virtual therapy service delivery. Recent calls have been made that the extension of universal design principles to the digital environment is essential in promoting equitable provision of digital services.^11^ Gaining insight into how telepractice functions in providing allied health services for people with disability and highlighting areas of potential improvement, has the potential to improve the universal design and accessibility for many population groups, including the elderly.^13^ However, questions remain regarding the delivery pathway characteristics required to achieve sustained and integrated uptake of telepractice.

Recent evidence has indicated an improvement in the efficacy and effectiveness of interventions provided via telepractice comparative to earlier iterations (through increased reliability and technological advancements)^14^, ^15^; and the coronavirus disease 2019 (COVID‐19) pandemic proved widespread uptake is possible.^16^, ^17^, ^18^ One aspect to consider in the pursuit of sustainable telepractice delivery is functionality, or whether telepractice works and how useful it is to end users.

How telepractice works and whether this aligns with the needs of users is a function of the form of telepractice in addition to context; in this case users include people with disability accessing allied health therapy interventions and their telepractice providers. Telepractice forms vary significantly from live videocall, to photographs or videos sent via email and online websites. This study's focus is synchronous videocall between clients and their provider, which was chosen as it provided real‐time visual and auditory communication for the purpose of therapy intervention.

Functional contextual factors include geographical location, disability support funding models, service distribution and the opportunity to choose between multiple service delivery options. The functionality and usefulness of telepractice for members of the disability community varies depending on their individual needs and barriers faced. However, if common threads of experience could be identified, there is the potential for sustainable integration of telepractice into service delivery models for people in the disability community. The primary research question addressed in this study was, how do allied health clinicians and managers perceive telepractice as functioning in the provision of therapy services to people with disability? The supporting subquestion was what influence does the provider/client relationship have on the perceived usefulness and subsequent adoption of telepractice for service delivery?

## 2 MATERIALS AND METHODS

### 2.1 Study design

The study is a qualitative review of experiences collected between November 2021 to February 2022 from disability organisations across Australia. Approval was gained from the Curtin Human Research Ethics Committee(ID# HRE2021‐0731) and reported in compliance with the Standards for Reporting Qualitative Research.^19^

### 2.2 Theory

Using qualitative methodology, a contextualist epistemological position^20^ was used, which assumes a coproduction of meaning by the participants and researchers that cannot be separated. Knowledge is viewed as a contextual representation of truth grounded in participant accounts, while acknowledging the role of the researcher.^21^, ^22^

The ontological stance taken utilises critical realism, which assumes a singular reality and retains the concept of truth while assuming an embedded influence of language and culture in each human experience, resulting in multiple perspectives and interpretations of this reality.^23^ Situated realities of participants are analysed by the researcher as findings which are located within their own subjective view of reality.^22^ The theoretical position aligns with the study aim as it enabled researchers to place the lived experience of participants centrally while considering the contextual and structural underpinnings of these accounts.^22^

Person‐first language conventions are utilised in deference to the preference of experts with lived experience who contributed to this research, to respect and affirm their identity. However, we respect the right to choose by participants and the potential for the alternate preference of identity‐first language by members of the disability community.^24^

### 2.3 Methods of data collection

Data collection included a Qualtrics demographic survey and semi‐structured interviews conducted and recorded via MS teams. The approach enabled in‐depth exploration of experiences across a wide geographical and jurisdictional area, within the context of travel limitations.

The semi‐structured interviews were guided by an interview schedule developed by the research team. Questions aligned to the salient constructs of the Consolidated Framework of Implementation Research (CFIR),^25^ and selected by a Steering Committee of staff and customers. The process was completed to ensure the questions and study focus were relevant to clients, service providers and the wider disability community. A copy of the interview schedule (Supporting Information: Appendix 1) and CFIR constructs (Supporting Information: Appendix 2) are provided as supplementary material. Interviews were transcribed via MS teams and reviewed for accuracy by C. B. Each participant was emailed a copy of their transcript with the opportunity to confirm validity and provide comment; feedback from four participants was included in the analysis.

### 2.4 Participant recruitment

Disability services are predominantly provided by large organisations which cater to many different service types. A consortium of 14 not‐for‐profit disability‐specific organisations called Ability First Australia (AFA)^26^ facilitated access to disability provider organisations representing the full scope of size and locations across the country. Each member of the AFA Consortium was offered an opportunity to participate in the study. For each eligible AFA organisation inclusion criteria were one manager involved in the design and implementation of telepractice and one therapist who delivered telepractice services. Staff whose role did not include providing direct therapy or management of therapy staff were excluded. Key informant and snowball sampling strategies within organisations were implemented during recruitment, which aimed to provide a variety of viewpoints and potentially identify differences through location, between roles and level of service provision. Participants were offered the option to select a pseudonym for publications, with all names replaced to safeguard anonymity.

### 2.5 Demographic characteristics

Fourteen AFA member organisations received invitations; eight responded positively, seven completed at least one interview (*n* = 13 interviews); six organisations and one manager participant did not respond. All participants were currently employed by an AFA organisation, English speaking, and consented to an interview. Demographics characteristics are included in Table 1 with geographical locations included all seven states of Australia excluding the two territories, three participants worked across two states and one participant worked from one state servicing another, both had moved interstate during the past year. Services were provided via telepractice before the COVID‐19 pandemic in a small‐scale capacity for rural clients in two organisations, with the remaining five providing none‐prior.

**Table 1:** Demographic characteristics.

Variable	*N* (%)
Gender (self‐identified)	
Male	0
Female	13
Not specified	0
Profession	
Behaviour support practitioner	1
Dietitian	0
Occupational therapist	3
Physiotherapist	3
Social worker	0
Speech pathologist	4
Nurse	1
Other	1
Role	
Management	6
Clinician	7
Location of service provision^a^	
New South Wales	2
Queensland	2
South Australia	2
Tasmania	2
Victoria	4
Western Australia	4

^a^ Three participants identified working across two states and one participant identified working from one state servicing participants in a different state (designated as the location of service rather than the location of staff member).

### 2.6 Data analysis methods

#### 2.6.1 Reflexive thematic analysis

Data analysis was conducted by the first author who engaged with the methodology of reflexive thematic analysis^27^ as it aligned with the goals of drawing patterns across the data set, a critical realist orientation and flexibility to describe core aspects within the data. Reflexive thematic analysis provided flexibility to integrate a metaphor method element to the naming of themes and description of findings.

#### 2.6.2 Metaphor analysis

The use of an analytical metaphor by the authors in reporting findings aimed to improve the accessibility of academic research for broader audiences, including the disability community. Metaphors are used as a way of structuring understanding of experiences,^28^ therefore can be utilised as a method of expanding understanding through linking familiar experiences to those less familiar or more complex. Recent examples in qualitative health research include a road trip in families with a Down Syndrome child,^29^ welfare systems as a pinball machine^30^ and an iceberg representing caring for ageing parents.^31^ In these examples and the current study, the authors selected a metaphor during the analytic process with the aim of improved understanding of complex topics reported in the findings^28^ and described with the use of symbols from the real world.^6^ This metaphor was selected through a collaborative process where lay responders were provided with multiple metaphor options in combination with the analytic themes to select the metaphor of best fit.

### 2.7 Analytic process

Data analysis commenced through building an understanding and familiarity while editing initial transcripts for accuracy. Initial codes were informed by the theoretical framework of the CFIR^25^ as it encouraged the first author in framing allocation to aspects of telepractice implementation. An inductive process was used to narrow themes and describe semantic and latent meaning from repeatedly reading initial codes.^22^

While reviewing themes, authors C. B. and J. D. discussed the fit of themes addressing core ideas produced by multiple tangential aspects of the experience of telepractice implementation. The phases of refining, defining and naming themes and subsequent writing were completed concurrently in a series of drafts which looked to incorporate the use of metaphor.

## 3 RESULTS

The exploration and analysis of interview data by the authors resulted in the identification of five themes relating to allied health provider perceptions of telepractice function and perceived usefulness. The five themes were derived from the data, and subsequently the research team proposed multiple metaphor options, from which lay responders selected *shopping for shoes* as the clearest visualisation that resonated with the findings. The five themes were named^1^ a shoe for every foot,^2^ planned purchases,^3^ shoe on the other foot,^4^ you need both shoes and^5^ help choosing their shoes.

### 3.1 A shoe for every foot

As people walk through life, they wear different shoes, be it comfortable shoes for walking, pretty shoes for fancy parties or the only pair they have. Every pair of feet are different, and for some people the two feet in their pair are different. As such, everyone has specific needs, limitations and considerations they factor into when, how and what shoes they wear. Accessing allied health services for people with disabilities is equally nuanced and individual in its considerations, as individual needs, capacity, desires and treatment options differ widely. The functionality of telepractice for delivering therapy services was thought to depend on a combination of factors, and might be perfect in some circumstances and impractical in others.

Participants emphasised the significance of age and life stage when assessing the suitability of telepractice and its integration into their client's overall life circumstances. Just as there are shoes you loved during childhood, but couldn't imagine wearing as an adult, telepractice can fit differently along life's journey. For example, older adults were described as enjoying clinic visits as a social outing providing human interaction, however, younger adults and adolescents found telepractice reduced the time burden of accessing therapy.

the clients that are more elderly, they see it as an outing. You know they've been stuck in the house all day and now they're going to their therapy session and it's like a little trip. [Clinician Megan]

Shoes with zippers or Velcro improve accessibility and can be beneficial additions for people who have difficulties in using shoes with laces. Telepractice, like a zipper, has the potential to accommodate the functional needs of individuals with disability, such as autistic people, people with mental health challenges or those who are respiratory compromised. Participants acknowledged that for some, telepractice is a preferred delivery mode and potentially the only achievable delivery mode.

Another customer I see she's got lots of mental health issues and for that reason she often just like refuses to see any professional and so I've only met her once in‐person. Every other session has been over telehealth and half the time she says no thanks. [Manager Jemma]

Conversely, for some clients, participants described telepractice exacerbating challenges like shoelaces that won't stay tied, including for those with physical difficulties or those who live in supported accommodation requiring support worker being present in the absence of their therapist. Challenges in completing sessions via telepractice were described based on a persons' support needs, as well as the capacity and dynamics of the wider support network. Participants raised concerns for families with additional challenges, including those whose first language was not English, similar to being given a pair of lace‐up shoes when all you've ever had were pairs with buckles, not impossible but significantly more challenging.

Other families were highlighted as having ‘chaos in my house’ [Manager Liz], with some perceiving increased stress of clients from therapists potentially seeing inside their home. These challenges were proposed as an indication of additional supports requirements for some families ‘logistics and the mental load’ [Adele] to enable telepractice to become more achievable and not judging them as lacking capacity.

This theme highlighted that as with specific types of shoes, telepractice may be the right fit for different people in different stages of their life or with different circumstances, and that some people may require a support person to assist them in creating that fit in accessing telepractice.

### 3.2 Planned purchases

Purchasing anything, including a pair of shoes without the proper preparation and resources can result in buyer's remorse. Participants described a functional telepractice session required access to resources for the videocall, assessments before the session and potentially the need to gather intervention‐specific resources.

Financial stress was highlighted by participants as a significant determinant of telepractice viability, which included access to necessary supplies and services to attend a videocall. Much as in the same way transport is a necessary cost to attend a shoe store in person, internet access is a major expense for clients and their families which is currently not supported by the NDIS.

some of our families from a low social economic background, there's a lot more barriers to it and so it's probably being less uptake, some of that is around, having access to Internet and that's often related to having credit on their phone. [Manager Danielle]

Several participants discussed solutions to the access limitations imposed by financial stress, with the NDIS enabling the purchase of an iPad or laptop, but not internet services for telepractice sessions. One clinician [Megan] described approaching charitable organisations to donate funds which supported an internet plan. Two participants described an initiative specific to a single Australian state supporting lower socioeconomic families at risk of poorer quality of life outcomes with internet and computer hardware access via child and family centres (CFCs). These CFCs provided government funding to improve equity in digital access opportunities for areas with higher levels of financially disadvantaged families, in the Australian state with the lowest gross state product per capita and a digital inclusion index five points lower than the national average.^32^ The clinician and manager both attributed the CFCs as a significant enabler within disadvantaged communities, and the clinician described a collaborative working relationship that supported families to see her virtually.

families when they have a telehealth appointment because they don't have the technology or Internet connection, they'll come into the centre … families learn how to access or use the technology and empower them that that they can do it themselves. [Clinician Shona]

Assessing clients is crucial for delivering effective and evidence‐based therapy sessions. Despite mixed opinions on conducting assessments via telepractice, participants generally agreed that in‐person initial assessments and establishing rapport were essential before remote sessions.

There are definitely limitations, and I found that much easier to transition to telecare once I had a good sense of the (child), I had evaluated him in‐person so much easier than to have those goals established. [Clinician Amina]

If circumstances required telepractice be used for initial assessments, Amina continued to say it was challenging but possible, similarly a person could measure their foot to order shoes online but generally find it easier in‐person.

Participants often described an increased mental load and time required to transition in‐person processes to virtual. Preparation time, such as sending resources or prompting families to gather specific items, was viewed as a positive it enabled practice outside direct therapy sessions. However, for some families this resource collection created additional financial stress or exclusion.

Completing a new task often causes increased time and effort in preparation and sourcing supplies, including potentially tailored supports for those experiencing additional barriers. However, once that first pair of shoes are purchased and the process is familiar, this knowledge enables each subsequent instance to become more efficient, and the same could be said for telepractice delivered sessions.

### 3.3 Shoe on the other foot

Most people require a pair of shoes consisting of a left and a right shoe, both shoes achieve the same result and are equally as useful but aren't the same. The distinct differences between left and right shoe improve their function, and as such if telepractice was considered the left shoe and in‐person service delivery was the right, the goal is the same however, the design is different. The fit of a pair of shoes may cause blisters on one foot but not the other, and similarly participants of this study identified both benefits and challenges of telepractice which were different to in‐person delivery.

A distinct difference of telepractice delivery is the different locations. Participants felt separation created reduced responsibility for therapists to carry out the actions of therapy and while empowering families to take on that responsibility. Participants described challenges in ‘trying to break those norms of bringing your child to therapy, we do therapy’ [Clinician Ella] with carers who weren't traditionally engaged and previously used the time as respite. Prior expectations derived from therapy delivered in‐person and perceiving telepractice as a direct equivalent decreased the likelihood of success, as highlighted by Shona comparing longer‐term clients:

changing expectations of how our sessions would look and their active need for participation probably was a bit of a barrier for some of the families. [Clinician Shona]

With new clients during the introduction of telepractice:

myself and one of the other speech therapists picked up some new clients during a time when we were only using teletherapy, and their expectation or their engagement in therapy was them being active participants. So, when we were able to see them face to face, that's how they naturally came into the sessions. That's all they knew. [Clinician Shona]

This comparison could be likened to only ever owning left shoes, with the potential that even if having a left and right shoe may improve comfort or function, a person could still prefer the familiarity of the original version. This comparison highlighted the differences and the need to provide realistic expectations before trialling telepractice for those who had historically experienced exclusively in‐person delivery. Additionally, it suggested the importance of introducing telepractice as a mode of delivery to new clients from the outset.

The active participation of families, which is encouraged by telepractice, was viewed positively by the majority of participants, with specific mentions of coaching interventions enabling capacity building and empowerment of families. One clinician described coaching and collaboration with disability support staff as a behaviour support clinician and found it to be very beneficial in viewing staff/client interactions in their natural environment:

So you do get a more authentic understanding of how the customer is behaving and what the nuances that are related to the staff and what they do. So I find it a fabulous way to get a really good understanding. [Clinician Margaret]

When considering a pair of shoes, the right shoe holds value to the right foot rather than being considered poor value to the left foot, and in parallel participants viewed telepractice as having distinct benefits separate to in‐person sessions. These benefits included group sessions for Key Word Sign (a sign language based on Auslan), bringing together sparce communities spanning vast geographical distances, enabling clinicians to view a clients' natural environment, and providing continuity of care for people who relocate frequently or seasonally.

Multiple managers discussed telepractice as adding another tool to the toolkit, whereas clinicians were more likely to discuss the need to learn to integrate telepractice as a new tool in future planning and normal practice. The prevailing sentiment remained that telepractice was a beneficial delivery option to provide flexibility in supporting clients to access services.

I think we've got a long way to go yet in this space and some work to do. But yeah, that's that would be my vision that it just becomes another option to use. [Manager Liz]

Left and right shoes excel at their roles, but could not replace the alternate shoe, participants equally perceived telepractice as existing to complement in‐person sessions and providing unique functions.

### 3.4 You need both shoes

Exclusively wearing right shoes would be challenging and not always result in comfortable feet, and similarly using telepractice exclusively for therapy delivery would not offer flexibility or the benefits of in‐person interaction. During the COVID‐19 pandemic a transition to full telepractice was required and demonstrated it is technically possible. You could also wear two right side shoes, however considering a long term sustained integration, participant sentiment indicated a hybrid is optimal.

Telepractice was commonly framed as an opportunity to increase flexibility and described as providing an alternate avenue for issues such as continuity of care, extended travel requirements, when a full hour session isn't appropriate, and three 20‐min sessions improves learning, and shorter check‐ins for consistency. When pairing the two delivery modes together clinician Shona reflected that goals were met at a higher frequency:

I've had experience with that with the clients that I've support remotely where we are doing a bit of a combination where I come out to their home … (for) face to face sessions and then we link in via telehealth and … it's worked really well and I'm finding that goals are being met more frequently with those families. [Clinician Shona]

This statement also highlighted the opportunity for outreach to remote communities, which can be especially challenging when multidisciplinary team engagement or senior staff input is required. Funding limitations can necessitate only one therapist travelling to remote communities or for singular clients, and telepractice provided the opportunity to include other team members in a consultation.

and I guess also as part of that, the ability to also have staff from other parts of the state consult into sessions. [Manager Danielle]

Telepractice was considered a useful avenue for providing services to clients on waiting lists due to staff shortages in their area. One manager described virtual staffing enabling interstate cooperation to meet client demand, similar to an online warehouse of a shoe retailer providing the desired shoes if the in‐person store is out of stock.

Clinicians and managers acknowledged that telepractice provided opportunities to improve care versus solely in‐person care but was not always the right fit. Examples of services that necessitate in‐person delivery included watching the dynamics of a student in their classroom, completing hip surveillance assessments and dysphasia swallowing assessments. Specific disciplines and tasks were viewed as better suited to telepractice than others, and a hybrid delivery was preferred.

Physio's and OTS desire to do things in person, being able to physically help a person to complete an exercise or to measure and be sure of wheelchair measurement or kitchen measurement and having that confidence behind it is totally different to the work of a speech pathologist. [Manager Jemma]

As the introduction to the theme implied, both is best in terms of left and right shoes and was advocated for by all participants in a hybrid model which empowers clients and clinicians to choose telepractice or in‐person delivery depending on the context.

### 3.5 Help choosing their shoes

When buying new shoes it is relatively common for people to seek advice and recommendations from trusted sources, be it their network of family and friends, social media influencers and advertisements. There are occasions where a person may not realise they are being influenced to buy one pair of shoes over another by the subconscious preferences of people around them. The theme ‘Help choosing their shoes’ addresses the potential sources for influence on the decision to access or avoid telepractice services by clients and motivators of influence from providers such as improving client quality of life or potentially more individual or organisationally derived origins.

Yeah … it was mostly a decision was made to move the whole organization to using Microsoft 365 and as part of that to use Teams. [Manager Danielle]

Discussions between clinicians and clients to utilise telepractice for funding reasons may be influenced by the opportunity to complete more therapy hours if travel is limited through digital delivery.

We found that, both therapist and customers weren't worried by doing telepractice, so they then started to address their transport levy…, they chose to take half of their sessions as telehealth so they didn't have to pay for 20 minutes travelled. [Manager Samantha]

The price of shoes purchased online or in store may be considered equivalent by salespeople, however incidental costs of attending the retail store may cause a cost disparity for customers. A participant's comment on direct cost to the client's funding plan without considering other associated costs indicated potentially ignored client savings:

I think other than you (the clinician) save on the travel costs. But I think really, it's the same cost if they came to our centre rather than doing it telehealth, it's going to cost them the same. [Clinician Ella]

Lacking consideration for what it would be like to walk in their (client) shoes and acknowledging potential travel costs to the clinic for families, loss of work time or child minding for other children, potentially fails to highlight benefits of telepractice. In contrast there are instances where participants described increasingly advocating for client choice and supporting those decisions be it telepractice or in‐person:

I think its longevity is growing, I think lots of families are preferring it and I haven't noticed that so much historically, and I felt like I have advocated it more, because the family safety and dynamics of who they are trying to keep safe lots of families are really choosing it. And then because they're seeing some benefits and how easy it can be. [Adele]

As with any purchasing of goods or services such as shoes or therapy, knowledge is power and customer purchasing power is crucial in supporting informed choice. Participants did not yet understand how this could be supported for all clients; however, they see the positive outcomes when telepractice as a true choice is achieved.

It was very much just the opportunity there was but as an organization we're really keen to continue to be able to offer it as a choice for clients and families. I think that would take a lot of work to embed. [Manager Liz]

## 4 DISCUSSION

The current study demonstrated that allied health clinicians and managers viewed telepractice as functioning best as an adjunct to in‐person sessions, using a hybrid delivery model. Provision of therapy services to people with disability was described as highly variable between individuals and at different points within an individual's life. A hybrid of telepractice and in‐person had the potential to cater to users as a plurality of different individuals with dynamic needs rather than one static universal individual, aligning with the concept of universal design.^7^ To digitise with purpose^33^ requires telepractice services designed not to be a direct repeat of in‐person delivery, and highlight distinct strengths and challenges linked to telepractice (*shoe on the other foot*, Section 3.3).

As demonstrated within the first theme (*a shoe for every foot*, Section 3.1) the functionality of telepractice was variable in relation to each context, with the level of usefulness potentially based on the correlation of individual need to the strengths of telepractice as a delivery mode. Findings by Gardner et al.^34^ supported the usefulness of telepractice for individuals who experienced challenges accessing the wider community due to increased anxiety and discomfort, a concept corroborated by participant Jemma in connection to an individual with significant mental health challenges. Articles by Daczewitz et al.^35^ and Hines et al.^36^ additionally uphold the significant usefulness of telepractice in facilitating flexible access to supplement in‐person therapy described in the theme *you need both shoes* (Section 3.4) in response to individual contexts of their specific cohorts, full time working parents and regional families.

Appropriate preparation and resourcing are required to improve the function and usefulness of telepractice for therapy delivery into the future, as advocated in the theme *planned purchases* (Section 3.2). The need to invest in equitable provision of connectivity^33^ has been called for in multiple academic and policy avenues, especially the gap between internet access for those with disability comparative to the wider community.^13^, ^37^ The Australian Government's Disability Strategy Outcomes framework^38^ directly references measuring the gap in digital inclusiveness between people with disability and the general community as a measure of focus.

Even with current levels of internet (including poor or limited access in regional areas) telepractice demonstrated its strength in delivering outreach services to supplement in‐person delivery for people in remote or under serviced areas in the theme *you need both shoes* (Section 3.4). Multiple published articles demonstrated similar success under the NDIS within Australia,^10^, ^36^ and internationally a study by Mitchell‐Gillespie et al.,^39^ utilised telepractice to redistribute educational opportunities into Africa, providing community rehabilitation to people from refugee populations.

Designing with a universal and accessibility approach has the potential to improve function and usefulness not just in telepractice for people with disability but for the wider community^13^; therefore, incorporating insights of people with disability should be prioritised as their thoughts and adaptations can help other groups who may face barriers to digital innovation. The final theme *help choosing their shoes* (Section 3.5) outlines the influential role providers can have in the decision‐making process to uptake services via telepractice or sustainably integrate telepractice in clients accessing therapy. If barriers to access are addressed and opportunities provided to facilitate universal access to telepractice for people with disability, the flow on effect would be increased usability for providers and therefore increase perceived usefulness and potential adoption of telepractice by clients. Recent investments by large technology companies such as Microsoft, Google and Amazon in programmes which prioritise improving accessibility of digital technologies,^13^ supports the impression that universal design and accessibility in the digital landscape is becoming a significant focus.

The onus of accessibility goes beyond the design of technological platform and features and extends to the need to support clinicians and clients through well‐designed services models. Workforce experience is one of the design principles for a people‐centred health system proposed by the Australian Consumer Health Forum. This health system proposal emphasises that to improve perceptions of telepractice usefulness and functionality for clients, clinicians require support.^33^ An article by Thomas et al.,^40^ highlighted that while person‐centred care was currently the predominant motivator for telepractice use, benefits beyond this are most likely needed in addition to improved integration into current workflows to support sustained client and clinician uptake. As clinicians hold a significant level of influence, they require support for capacity building and positive telepractice experiences (*help choosing their shoes*, Section 3.5), a sentiment echoed across respondents in South Asia, Kuwait and Europe in the article by Oommen et al.^41^ Good service design principles as outlined by Downe,^42^ describe the need for a service which requires no prerequisites to access, linking to the need for telepractice delivery services to include support and upskilling for clients within the design. As outlined in the first theme (*a shoe for every foot*, Section 3.1) individuals in different contexts will have varying requirements to remove barriers to equitable access, and as argued by Chapman et al.^43^ the onus of competency and resilience should not be the responsibility of the individual, potentially exacerbating levels of inequality.

## 5 LIMITATIONS AND FUTURE DIRECTIONS

The analysis describes a snapshot of time and perspectives of participants who were reflecting on past and present experiences with telepractice, with the COVID‐19 pandemic occurring ongoing during the interviews and participants completing interviews over a 4‐month period. Timing of each interview and the order completed may have impacted on reflections of each. The challenges of completing research during a pandemic created uncertainty, but additionally depicts the reality of participants in their context and associated challenges.

The transferability^44^ of findings specific to experiences of participants and study context may be limited in direct comparison to alternate locations, contexts and times, however, the reader may find opportunities to extrapolate the findings to guide future telepractice policy, implementation or investigations. As the theoretical position of the study assumes multiple interpretations of reality created by each participant's experience and described through the lens of the researcher, a limitation exists in the singular perspective of participants in provider roles. The distinction is critical in the interpretation of findings, with publications such as Barkai et al.,^45^ highlighting a significant divergence between provider and client experiences in the context of virtual service delivery. The divergence in experience, combined with perspective pieces such as Kendall et al.,^11^ and Noel et al.,^13^ which advocate for the inclusion of people with a disability's voices, indicates a strong need to ask clients directly and build understanding of their lived experience. As a future direction, the exploration of client voices could work to further strengthen the findings of the current study.

Technological innovation offers an obvious opportunity for future advancements in functionality and usefulness of telepractice. For technological innovations to be implemented requires awareness of technology capabilities potentially facilitated through linking of disability and technology sectors. Technology design is only part of the solution to reduce the burden of accessibility and as such service design must equally prioritise facilitating universal design. Future investigation into the most appropriate strategies to facilitate design of these services in partnership with clinicians and clients is required.

## 6 CONCLUSION

Consciously improving the functionality and usefulness of telepractice in a universally accessible manner has the potential to improve experience for innumerable users not limited to those with a disability. A strategy for improving perceived usefulness as a service delivery mode may involve positioning telepractice as a unique delivery mode with strengths and weaknesses, not as a replacement for in‐person care. the adaptability and variability of a hybrid of telepractice and in‐person service delivery has the opportunity to support the individuality of people's needs rather than striving for one singular optimal digital solution. these findings support the perception of participants that telepractice functions best as an adjunct to in‐person sessions through a flexible hybrid delivery model in the provision of therapy services to people with a disability

## AUTHOR CONTRIBUTIONS

**Cloe Benz**: Conceptualisation; methodology; investigation; writing—original draft; visualisation; formal analysis. **Jaya Dantas**: Validation; writing—review and editing; supervision. **Mai Welsh**: Conceptualisation; project administration; writing—review and editing; supervision. **Richard Norman**: Conceptualisation; supervision; writing—review and editing. **Suzanne Robinson**: Conceptualisation; writing—review and editing; supervision. **Delia Hendrie**: Conceptualisation; writing—review and editing; supervision.

## CONFLICT OF INTEREST STATEMENT

Cloe Benz, Jaya Dantas, Richard Norman, Delia Hendrie and Suzanne Robinson report no conflicts of interest with regard to this paper. Mai Welsh at the time of the study, was employed by Rocky Bay a Not‐For‐Profit Disability Service provider who function as the industry partner for the project. All co‐authors have seen and agree with the contents of the manuscript and there is no financial interest to report. The authors certify that the submission is original work and is not under review at any other publication.

## ETHICS STATEMENT

The study was approved by the Curtin University Human Research Ethics Committee (ID# HRE2021‐0731). All participants of the study provided written informed consent before participation.

## Supporting information

Supporting information.

## Data Availability

The data that support the findings of this study are available on request from the corresponding author. The data are not publicly available due to privacy or ethical restrictions.
