# Supplementary material for: A qualitative study assessing allied health provider perceptions of telepractice functionality in therapy delivery for people with disability
Source: Health Expect. 2024 Feb 13;27(1):e13988. doi: 10.1111/hex.13988 (PMC10864922; doi:10.1111/hex.13988)
Supplement: Supplementary file 1 — Supporting information. [file HEX-27-e13988-s001.docx]

**A qualitative study assessing Allied Health Provider perceptions of telepractice functionality in therapy delivery for people with disability**

Supplementary Material

**Appendix 1:**

| Topic | Key Question | Follow-up questions |
| --- | --- | --- |
| Introduction and implementation of the intervention | Can you describe the introduction of telepractice at your organisation?  What were some positives that came out of implementing Telepractice? | What was your role in the telepractice introduction?  How prepared did you feel at the time of implementation?  Did you have sufficient resources? What investments did you need to make? (put into organisation section)  What was the initial response for?  How does telepractice compare to usual care? |
|  | What videoconferencing platform/s do you use and why? | Do you offer a choice of videoconference platforms to your staff/customers?  What are your preferences and why?  What risks are there with the platform that you use and how do you mitigate these? |
|  | What were the main barriers and facilitators you or your clients faced to implementing Telepractice? | What were some positives that came out of implementing Telepractice?  Are there differences in quality between telepractice and usual care?  Are there differences in cost between telepractice and usual care?  What strategies did you put in place to address these barriers? |
|  | What kind of resources and supports were required for Telepractice implementation? | Do you feel there were additional resources that you required and didn’t have?  Who was responsible for the resourcing, training, and support for the telepractice program? Was it timely, relevant, and sufficient? |
|  | What has changed and/or how have you adapted since the initial implementation of Telepractice? | Have you made any disability specific modifications to usual telepractice sessions? |
|  | Reflecting on this process, what would you recommend to someone starting out? | How have you reflected and evaluated in your organisation on the success of telepractice implementation?  What would you recommend as something you would do, vs things you wouldn’t do? |
| External Contexts and Patient needs | How do you feel the Pandemic and related government/health mandates have impacted on your use of Telepractice? | Did the pandemic have a direct impact on your decision to implement telepractice, either through forcing the introduction or speeding up the timeframe?  How do you feel the length of time you were in lockdown due to the pandemic changed or impacted on the telepractice service?  Is there a noticeable difference in uptake of telepractice during lockdown periods vs normal business? |
|  | Do you feel the NDIS adequately supports the use of telepractice in the disability sector? | Did you facilitate purchase of technology or specific training to enable telepractice sessions? How did you do this? (funding)  Do you feel the use of telepractice supports the principle of choice and control for users of the NDIS? |
|  |  | (CLINICIANS) Did your therapy discipline professional registration body provide education and support to guide your practice during the transition to virtual delivery? |
|  | How have your customers responded to the transition to telepractice? | Do they feel it meets their needs?  Have you requested feedback from customers regarding telepractice?  How are the needs of customers met through telepractice? |
| Characteristics of the organisation & Individual using Telepractice | Could you please describe the culture of your organisation in implementing change? | What else was happening at the time? Do you feel this impacted on the success or openness for change of the staff and service users?  Did you feel there was/is clear goals and feedback provided by the organisation regarding telepractice? |
|  | Do you perceive notable differences in motivations to use telepractice from specific groups? | Are there notable differences in providing telepractice services to metropolitan vs regional customers?  Do you feel you or your staff, and customers feel confident in using telepractice to provide/engage in services?  Is there a notable difference between initial session vs subsequent follow up appointment? |
|  | Do you feel your organisation was open to the introduction of telepractice? | Were there biases towards or against specific groups of clinicians or clients using telepractice? E.g. elderly customers, specific therapy disciplines etc.  Has that changed from initial implementation to now?  Are there specific kinds of consults and specific kinds of clients which will keep using telepractice into the future? |
| Closing Question | All things being equal, with any kind of advancements you can think of; what do you see as the future for this type of technology/ service delivery? |  |

**Appendix 2:** Consolidated Framework For Implementation Research^1^ – Salient Constructs

| **Domain** | **Salient Construct** |
| --- | --- |
| Intervention | Relative Advantage  Adaptability  Complexity  Cost  Design Quality and Packaging |
| Outer Setting | Patient Needs and Resources  External Policy and Incentives |
| Inner Setting | Culture  Compatibility  Learning Climate  Available Resources  Goals and Feedback  Implementation Climate  Access to Knowledge and Information |
| Individual | Knowledge and Beliefs  Personal Attributes  Individual Stage of Change  Self-Efficacy |
| Implementation | Engaging  Reflecting & Evaluating |
| 1. Kirk MA, Kelley C, Yankey N, et al. A systematic review of the use of the Consolidated Framework for Implementation Research. *Implement Sci* 2016; 11: 72-72. DOI: 10.1186/s13012-016-0437-z. | |
